# Changes in Soil Physico-Chemical and Microbiological Properties During Natural Succession: A Case Study in Lower Subtropical China

**DOI:** 10.3389/fpls.2022.878908

**Published:** 2022-06-03

**Authors:** Xinyu Zhao, Peiling Liu, Yingjie Feng, Weiqiang Zhang, Brian Njoroge, Fengling Long, Qing Zhou, Chao Qu, Xianhua Gan, Xiaodong Liu

**Affiliations:** ^1^College of Forestry and Landscape Architecture, South China Agricultural University, Guangzhou, China; ^2^South China Botanical Garden, Chinese Academy of Sciences, Guangzhou, China; ^3^Guangdong Provincial Key Laboratory of Silviculture, Protection and Utilization, Guangdong Academy of Forestry, Guangzhou, China

**Keywords:** forest restoration, physico-chemical properties, microbes, subtropical forests, soil quality index, soil stoichiometric ratio

## Abstract

Vegetation succession can change the function and quality of the soil. Exploring the changes in soil properties during secondary forest restoration is of great significance to promote forest restoration and improve the ecological service function of subtropical ecosystems in South China. In this study, we chose three typical forests in subtropical China as restoration sequences, broadleaf–conifer mixed forest (EF), broad-leaved forest (MF), and old-growth forest (LF), to study the changes in soil physico-chemical and biological properties and the changes of soil comprehensive quality during the secondary succession of subtropical forest. The results showed that the soil physical structure was optimized with the progress of forest succession. The soil bulk density decreased gradually with the progress of forest restoration, which was significantly affected by soil organic carbon (*p* < 0.01). In LF, the soil moisture increased significantly (*p* < 0.05), and its value can reach 47.85 ± 1.93%, which is consistent with the change of soil porosity. With the recovery process, soil nutrients gradually accumulated. Except for total phosphorus (TP), there was obvious surface enrichment of soil nutrients. Soil organic carbon (15.43 ± 2.28 g/kg), total nitrogen (1.08 ± 0.12 g/kg), and total phosphorus (0.43 ± 0.03 g/kg) in LF were significantly higher than those in EF (*p* < 0.05). The soil available nutrients, that is, soil available phosphorus and available potassium decreased significantly in LF (*p* < 0.05). In LF, more canopy interception weakened the P limitation caused by atmospheric acid deposition, so that the soil C:P (37.68 ± 4.76) and N:P (2.49 ± 0.24) in LF were significantly lower than those in EF (*p* < 0.05). Affected by TP and moisture, microbial biomass C and microbial biomass N increased significantly in LF, and the mean values were 830.34 ± 30.34 mg/kg and 46.60 ± 2.27 mg/kg, respectively. Further analysis showed that total soil porosity (TSP) and TP (weighted value of 0.61) contributed the most to the final soil quality index (SQI). With the forest restoration, the SQI gradually increased, especially in LF the value of SQI was up to 0.84, which was significantly higher than that in EF and MF (*p* < 0.05). This result is of great significance to understanding the process of restoration of subtropical forests and improving the management scheme of subtropical secondary forests.

## Introduction

Forests are universally known to be one of the most important terrestrial ecosystems in terms of the diverse ecological functions they serve, including carbon sequestration, biodiversity conservation, water, soil conservation and cleaning the air, etc. (Jim and Chen, 2009; Song et al., 2016; Kuehler et al., 2017). A serious environmental crisis has been caused by the reduction of forest area thus, forest restoration has gained a lot of attention from governments and ecologists (Moreno-Mateos et al., 2017). In recent years, ecosystem recovery which is proposed to solve the degradation issues has increasingly occurred worldwide (Moreno-Mateos et al., 2017).

Forest restoration has been documented to be accompanied by changes in the species’ structure and soil evolution (Prestes et al., 2020). Over the past few decades, a large number of studies have emerged on the succession process of subtropical secondary forests. It was reported that the diversity of species and canopy increased during the development of forest phytocenoses, together with the enhancement of the connection between populations (Bruelheide et al., 2011; Zhang et al., 2021). In addition, those changes in forest vegetation mentioned above also affect the amount of solar radiation reaching the forest floor (Dietz et al., 2020; Onoda and Bando, 2021). The inter-relationship between the vegetation and climate factors forms distinctive microhabitat conditions in different forests, which alters soil properties by affecting the process of litter decomposition and root development, and further impacts the coupling relationship of soil C-N, C-P, and N-P. According to interactive soil–vegetation feedback, the plant communities improve the soil structure and fertility, which ulteriorly allows the community to develop (Brooker et al., 2015; Chai et al., 2019). There is a natural coupling relationship between soil and vegetation systems, which is an important mechanism of forest development and succession, especially on a small spatial scale (van der Putten et al., 2013; Huang et al., 2018). In other words, forests in different succession stages influence soil status to various magnitudes (Pang et al., 2018).

Pedosphere is an active interface in the biogeochemical cycle of forest ecosystems, and its physical, chemical, and microbial characteristics can indicate the process of soil formation, soil development, and fertility along forest succession (Chen et al., 2019a). Soil quality index (SQI) can be used as a parameter that integrated various physical, chemical, and biological indexes to judge the development of soil, and find out the main controlling factors affecting the change of soil, so as to facilitate future operation and management. Shao et al. (2020) reported that water holding capacity, soil organic matter, total nitrogen, available potassium, and altitude appeared to be the main limiting factors of soil quality of Mount Tai, China. Muñoz-Rojas et al. (2016) found that in addition to organic carbon and C:N ratio, biological indicators are the most sensitive indicators to detect differences among soils in semiarid areas. Currently, few relevant studies have been conducted with respect to the impact of secondary forest restoration on soil properties in subtropical China, which limits the overall evaluation of soil quality in subtropical areas.

Xinfengjiang National Forest Park is located in Guangdong Province, China, and it includes forest types in different succession stages. Due to extensive deforestation in the 1950s, most of the stands in the study site are now secondary forests (Zhang et al., 2019b). In 1984, the Chinese government promulgated a new forest law, which implemented quota cutting for forests and encouraged afforestation. Since then, deforestation was prohibited in this study area and the forests here were allowed to gradually undergo self-succession in the three decades that followed. As those forests experienced intensive human disturbance and strict protection, they can be regarded as a suitable system to study the changes in soil properties along with the restoration in subtropical secondary forests. We selected three types of forests, named broadleaf–conifer mixed forest (EF), broad-leaved forest (MF), and old-growth forest (LF), which represent a typical forest restoration sequence in subtropical China. In this paper, we assume that the overall soil quality will be improved to a certain extent with forest succession. We intend to provide answers to the following problems. Firstly, how do soil physico-chemical properties and biological properties change along with forest succession? Secondly, what is the relationship between different soil properties? Thirdly, in the process of forest succession, what are the main controlling factors affecting the change of the soil? Our findings may provide references for sustainable forest management in South China.

## Materials and Methods

### Site Description

The study area is located in the Xinfengjiang National Forest Park (23°40′30″–24°46′39″ N, 114° 30′33″–114°36′30″ E), China, with a total area of 4479.47 hectares. The region has a subtropical monsoon climate, with a forest coverage rate of 78%. The vegetation types are mainly an evergreen broad-leaved forest, an evergreen coniferous forest, and a broadleaf–conifer mixed forest. The average temperature is 21.2°C and the annual average precipitation is about 1,420 mm, mainly from April to September, and the average relative humidity is about 76%. Bedrocks are classified as granite, basalt, and sandshale. Soils are classified as yellow and red earth, mostly medium loam and heavy loam (Huang et al., 2018).

**Figure 1:**
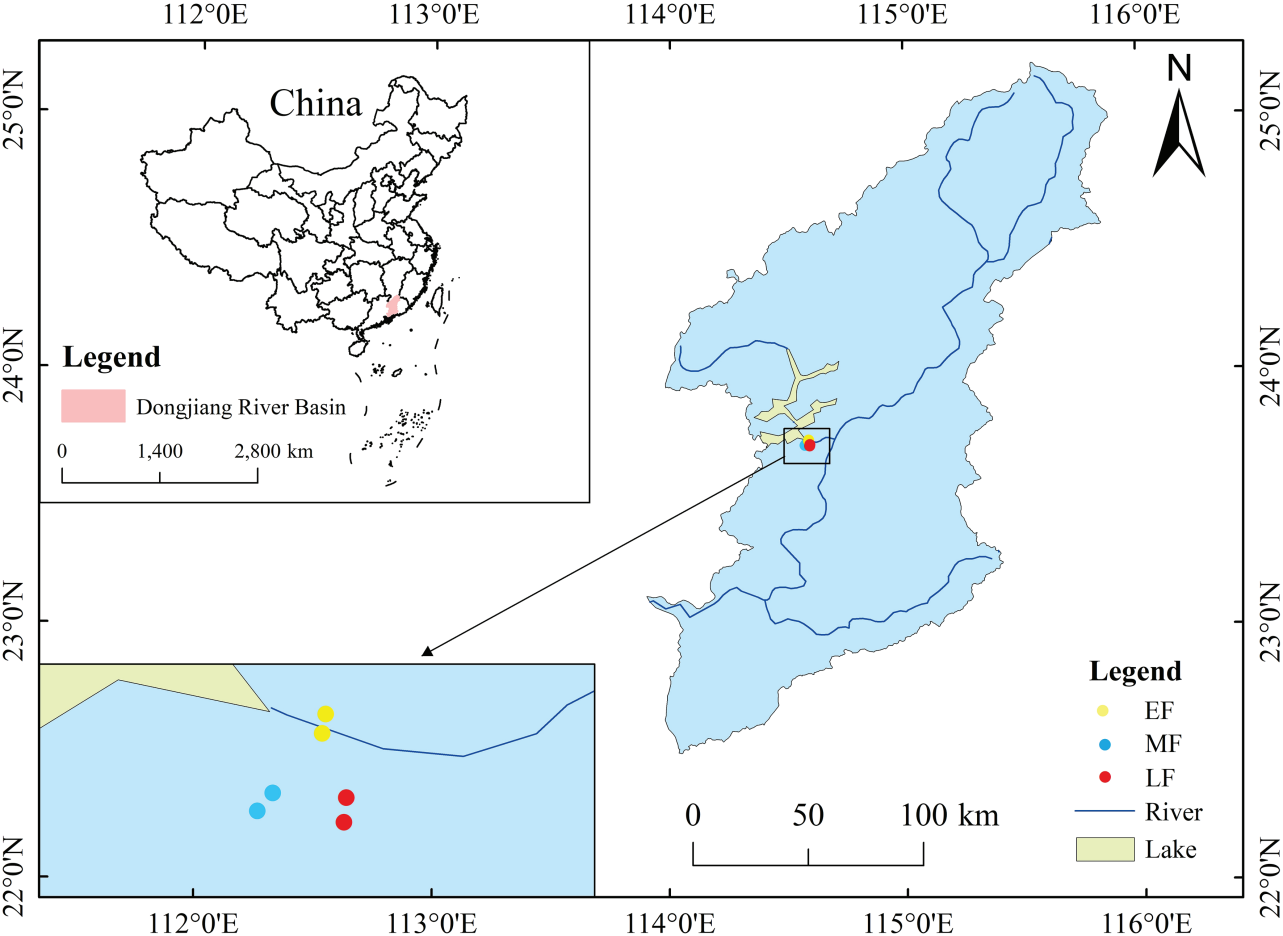
Location of the study area at the Xinfengjiang National Forest Park, Guangdong Province, China.

In 2014, six plots were permanently set up in the study area, of which two duplicate plots were established for each stand type (Figure 1). Each plot with an area of 50 m × 50 m was close to the basic factors, such as altitude and slope. The selected plots covered three different types of forests in the area: a broadleaf–conifer mixed forest (EF), a broad-leaved forest (MF), and an old-growth forest (LF), which represented a sequence of forest restoration based on tree species composition and the degree of interference (Huang et al., 2018). The broadleaf–conifer mixed forest was developed from coniferous tree species, and the dominant tree species included *Pinus massoniana* and *Cunninghamia lanceolata*. The broad-leaved forest was partially logged before conservation, and the dominant tree species are *Castanopsis chinensis*, *Schefflera octophylla*, and *Itea chinensis*. The old-growth forest is relatively close to the central area and has not been disturbed much in the past, so it can be regarded as a well-developed forest in the later stage of succession. Its dominant tree species are *Schima superba*, *Cinnamomum porrectum*, and *Machilus thunbergii*.

### Soil Sampling

In every plot, three randomly repeated small quadrats were set up with an area of 10 m × 10 m. We dug two holes about 1.3 m × 1 m in size for collecting soil samples in each quadrat. Soil samples were taken at 0–25 cm, 25–50 cm, 50–75 cm, and 75–100 cm layers for the determination of the soil’s physical and chemical properties. The soil microbial biomass was determined using the samples taken at 0–30 cm, 30–60 cm, and 60–90 cm layers. Soil sampling was conducted once during the rainy season and dry season, respectively, a total of 66 samples were collected (24, 24, and 18 for the determination of physical, chemical properties, and microbial biomass, respectively) each time for each stand. Three duplicate undisturbed soil cores were collected with 100 cm^3^ stainless steel cylinders from each soil layer. The disturbed soil samples were collected using a soil drill with a diameter of 3.5 cm from five points, at the four corners and the center of each quadrat, and then the samples from the five points in each layer were fully mixed making up the composite samples (the weight of each composite sample was about 1 kg). The fresh soil samples from every 30 cm were stored in a 4°C refrigerator for the determination of soil microbial biomass C (MBC) and microbial biomass N (MBN).

### Measurements

The bulk density (BD), soil water content (SWC), and porosity were determined using the undisturbed soil sample cores using Kopecky’s cylinder method (Piaszczyk et al., 2020; Grzywna and Ciosmak, 2021). We first recorded the weight of the empty cylindrical soil auger with a volume of 100 cm^3^ (m_0_). After sampling, we weighed the cylindrical soil auger with the contained fresh soil sample (m_1_). We then soaked the cylindrical metal core with the undisturbed soil in water for 12–14 h and weighed it thereafter (m_2_). The specific calculation formula used is as follows:


(1) $$md=\left(m_{1}–m_{0}\right)\times \left(1–SWC\right)$$

(2) $$MWHC=\left(m_{2}–md–m_{0}\right)/md\times 100$$

where *SWC* is water content of soil (%); *md* is the dry soil weight (g); *MWHC* is the maximum water holding capacity (%).

Plant roots, debris, and gravels were removed before the analysis of the mixed soil, and the soil samples from every 25 cm were screened through a 2-mm mesh for available phosphorus (AP) and available potassium (AK); through a 1-mm mesh for soil alkaline hydrolysis nitrogen (AN); and through a 0.25-mm mesh for soil organic matter (SOM), total nitrogen (TN) and total phosphorus (TP; Chen et al., 2020). The SOM was quantified using the potassium dichromate oxidation method (Dong et al., 2018; Yang et al., 2020). The van Bemmelen factor (1.724) was used for conversion between SOM and SOC. The total nitrogen (TN) amount was measured using the Kjeldahl digestion method (Cao et al., 2020). The content of TP was determined using hydrochloric acid, hydrofluoric acid, and nitric acid digestion-inductively coupled plasma atomic emission spectrometry (ICP-AES; He et al., 2021). The content of AN was measured by the alkali-hydrolysis and diffusion method (Cui et al., 2018). The amount of AP was detected using a spectrophotometer (Wu et al., 2019). The content of AK was quantified using a flame photometer after the samples had been prepared using an ammonium acetate solution (Wu et al., 2019). The contents of MBC and MBN were determined by the chloroform fumigation extraction method (Dong et al., 2018).

### Evaluation of Soil Quality Index

We selected a soil quality index (SQI) to comprehensively represent all the indicators we had measured to evaluate the overall soil quality. It has been widely used under the condition of minimum data sets (MDS), which enables researchers to select the most representative soil indicators and reduce data redundancy (Zhang et al., 2019a). The establishment of SQI mainly includes three steps: (1) selecting appropriate indicators to build MDS, (2) calculating the MDS index score and determining the index weight, (3) combining the index scores into SQI.

The principal component analysis method was used to screen MDS, select the principal components with values greater than 1, and rotate the maximum variance of the selected principal components to enhance the interpretability of irrelevant components (Li et al., 2013). In each selected principal component, the index with the absolute value of the selected factor load within 10% of the maximum factor load was the high factor load index (D’Hose et al., 2014). When only one high factor load index was left in the main component, the index enters MDS; when more than one high factor load index was left in the main component, Pearson’s correlation analysis was used to check whether other indexes should be deleted. If the correlation coefficient *r* < 0.7, all indexes were selected into MDS; if the correlation *r* > 0.7, the high factor load index with the largest sum of correlation coefficients was selected into MDS (Li et al., 2013; Zhang et al., 2019a). After determining the indicators of MDS, a non-linear scoring function was performed to transform the soil indicators into scores that ranged from 0 to 1. The sigmoidal function [Eq. (3)] was used as follows (Ciarkowska and Gambus, 2020):


(3) S=a1+xx0^b

Where *S* is the score of soil indicator, *a* is the maximum score (*a* = 1) reached by the function, *x* is the value of the indicator, *x_0_* is the mean value of each soil indicator, and *b* is the value of the equation’s slope (Zhang et al., 2019a). Slope values (*b*) of −2.5 was set for “more is better” and 2.5 was set for “less is better” curve, respectively (Ciarkowska and Gambus, 2020). Weighting values for every indicator were assigned by commonality which was calculated using factor analysis (FA; Rahmanipour et al., 2014). Finally, with the scores and weighting values, SQI was calculated using the following equation [Eq. (4); Zhang et al., 2019a]:


(4) $$\mathrm{SQI}=\overset{n}{\underset{i=1}{\sum }}S_{i}*W_{i}$$

Where *W_i_* is the weighting value of the selected soil indicators determined by PCA, *S_i_* is the indicator score calculated by Eq. (3), and *n* is the number selected in MDS.

### Statistical Analysis

Before statistical data analysis, the Kolmogorov–Smirnov test (*p* = 0.05) was performed on the experimental data and the normality of the data was judged. One-way ANOVA was used to compare the differences in soil properties between different vegetation types and soil depths. When the ANOVA results were significant according to the *F* value (*p* < 0.05), Duncan’s test was performed to compare the mean differences of soil variables. All sample mean values and standard errors in this study were processed and analyzed by Microsoft Excel 2010 (Microsoft Corporation, Redmond, WA, United States) and IBM SPSS Statistics 22.0 (International Business Machines Corporation, Armonk, NY, United States), and the data results were plotted using Origin 2021 (Originlab Corporation, Northampton, MA, United States).

## Results

### Differences in the Soil Physico-Chemical Properties of the Three Forests

As shown in Table 1, the BD increased with increasing soil depth in different forests, and the BD of the surface layer was significantly lower than that of other soil layers. The BD for the 0–100 cm depth gradually decreased along forest restoration, that is, EF (1.33 ± 0.03 g/cm^3^) > MF (1.28 ± 0.03 g/cm^3^) > LF (1.16 ± 0.03 g/cm^3^). The TSP decreased with increasing soil depth gradually in EF and MF, while the change in TSP was not significant between layers in LF. When averaged across the 0–100 cm depth, the TSP value was significantly decreased in the order LF (52.36 ± 0.85%) > MF (45.53 ± 1.17%) > EF (42.41 ± 0.85%). In this study, soil porosity in three forests consisted mainly of capillary porosity. As forest restoration continued, the CP increased significantly from 33.63 ± 0.93% (EF) to 48.77 ± 0.99% (LF). When averaged across the 0–100 cm depth, the NCP in EF (8.79 ± 0.72%) was significantly higher than that in MF (4.61 ± 0.48%) and LF (3.60 ± 0.54%). The MWHC was regarded as the amount of water held by the soil when all its pores are filled with water. In each forest, the MWHC gradually decreased with increasing soil depth. When averaged across the 0–100 cm depth, the MWHC (47.85 ± 1.93%) in LF was significantly higher than that in other forests (about 32.63 ± 1.39% in EF and 37.27 ± 1.59% in MF, respectively).

**Table 1:** The soil’s physical characteristics across the 0–100 depth.

	Soil layer (cm)	BD (g/cm^3^)	TSP (%)	CP (%)	NCP (%)	MWHC (%)
EF	0–25	1.12 ± 0.05Ba	46.37 ± 1.13Aa	35.36 ± 2.39Ab	11.01 ± 1.67Aa	41.80 ± 1.27Ab
	25–50	1.33 ± 0.02Aa	43.38 ± 1.28ABb	34.67 ± 1.70Ac	8.71 ± 0.73Aa	32.77 ± 1.37Bb
	50–75	1.44 ± 0.04Aa	41.08 ± 1.70BCb	34.23 ± 1.64Ab	6.86 ± 1.65Aa	28.84 ± 2.05BCb
	75–100	1.44 ± 0.04Aa	38.83 ± 1.08Cc	30.26 ± 1.24Ac	8.57 ± 1.30Aa	27.13 ± 1.38Cb
MF	0–25	1.13 ± 0.04Ba	47.77 ± 2.00Aa	41.78 ± 2.68Aab	6.00 ± 0.95Ab	42.64 ± 2.68Ab
	25–50	1.29 ± 0.05ABa	46.57 ± 1.69Ab	42.27 ± 2.24Ab	4.30 ± 0.72Ab	36.56 ± 2.13Ab
	50–75	1.38 ± 0.08Aab	44.18 ± 3.24Ab	40.58 ± 3.81Ab	3.59 ± 0.73Aab	34.80 ± 3.78Ab
	75–100	1.34 ± 0.06Aab	45.09 ± 2.56Ab	40.56 ± 3.68Ab	4.54 ± 1.32Ab	35.36 ± 3.44Aa
LF	0–25	1.01 ± 0.08Ba	52.54 ± 3.15Aa	45.14 ± 3.09Aa	7.40 ± 0.90Ab	60.71 ± 3.66Aa
	25–50	1.13 ± 0.02ABb	52.81 ± 0.87Aa	50.27 ± 1.01Aa	2.54 ± 0.35Bb	46.73 ± 1.37Ba
	50–75	1.23 ± 0.03Ab	52.39 ± 0.98Aa	50.16 ± 1.45Aa	2.23 ± 0.65Bb	42.87 ± 1.71Ba
	75–100	1.26 ± 0.03Ab	51.70 ± 1.17Aa	49.49 ± 1.41Aa	2.22 ± 0.47Bb	41.09 ± 1.73Ba

*BD, bulk density; MWHC, maximum soil water holding capacity; TSP, total soil porosity; CP, capillary porosity; NCP, non-capillary porosity; EF, broadleaf–conifer mixed forest; MF, broad-leaved forest; LF, old-growth forest. Lowercase letters represent statistically significant differences among different stand types in the same soil layer. Capital letters represent statistically significant differences among different soil layers in the same stand type (Duncan’s tests, p < 0.05)*.

### Differences in the Soil Nutrient Contents of the Three Forests

**Figure 2:**
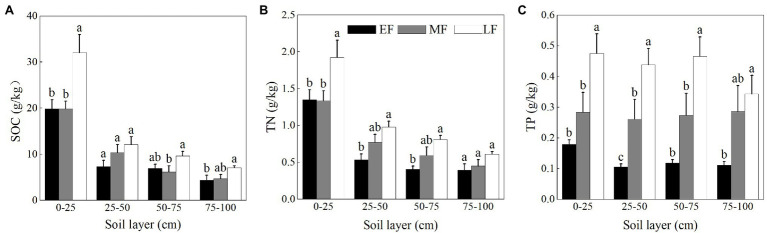
The contents of SOC **(A)**, TN **(B)**, and TP **(C)** in the 0–100 cm soil layer of different forests. Values are the means ± SE (*n* = 6). Lowercase letters above the columns represent statistically significant differences among stand types for the same soil layer (Duncan’s tests, *p* < 0.05). SOC, soil organic carbon; TN, soil total nitrogen; TP, soil total phosphorus; EF, broadleaf–conifer mixed forest; MF, broad-leaved forest; LF, old-growth forest.

As shown in Figures 2A,B, the content of SOC and TN of each forest showed significant surface enrichment in the soil profile. The SOC content of the surface layer (0–25 cm) increased significantly alongside forest restoration with values ranging from 19.83 ± 1.69 g/kg to 32.03 ± 3.93 g/kg, and the TN content of LF was highest (1.92 ± 0.24 g/kg) among forests. When averaged across the 0–100 cm depth, the SOC content between forests was in the order LF (15.43 ± 2.28 g/kg) > MF (10.27 ± 1.40 g/kg) > EF (9.64 ± 1.41 g/kg), and the TN content increased from 0.67 ± 0.09 g/kg (EF) to 1.08 ± 0.12 g/kg (LF). As shown in Figure 2C, the variation of the TP content in the vertical section was relatively stable. The TP content for the 0–100 cm depth between forests increased in the order EF (0.13 ± 0.01 g/kg) < MF (0.28 ± 0.03 g/kg) < LF (0.43 ± 0.03 g/kg).

**Figure 3:**
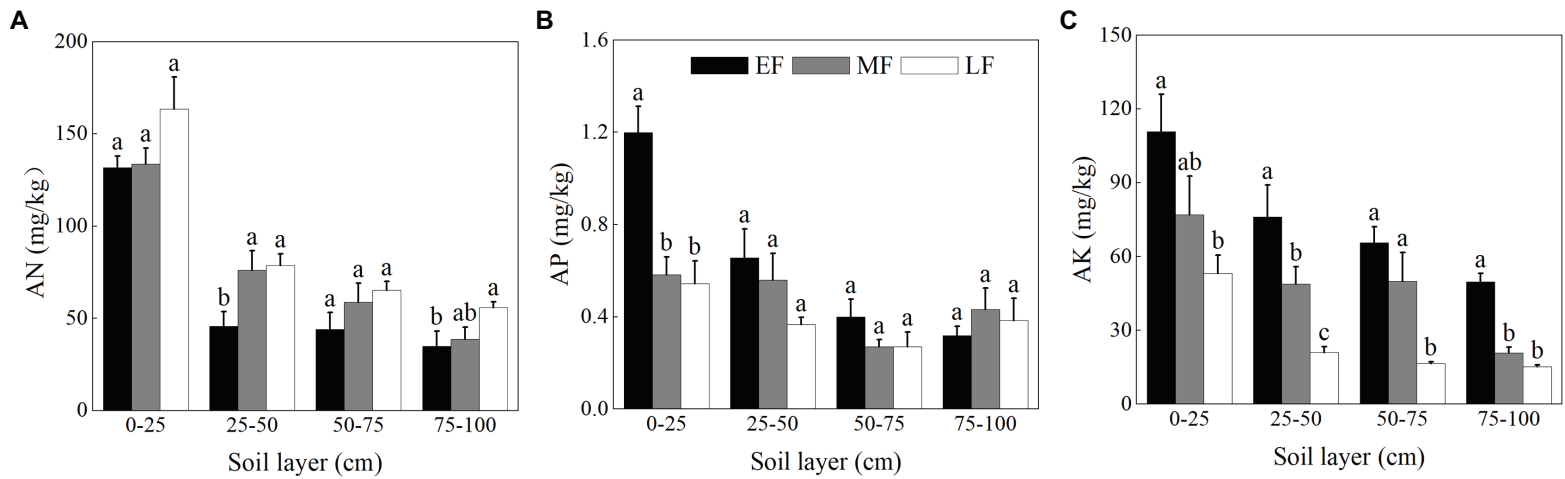
The contents of AN **(A)**, AP **(B)**, and AK **(C)** in the 0–100 cm soil layer of different forests. Values are the means ± SE (*n* = 6). Lowercase letters above the columns represent statistically significant differences among stand types for the same soil layer (Duncan’s tests, *p* < 0.05). AN, alkali-hydrolyzable nitrogen; AP, available phosphorous; AK, available potassium; EF, broadleaf–conifer mixed forest; MF, broad-leaved forest; LF, old-growth forest.

As shown in Figure 3A, the AN content in the surface soil (0–25 cm) was significantly higher than that of the other soil layers in each forest. When averaged across the 0–100 cm depth, the AN content was ranked as LF (90.85 ± 9.98 mg/kg) > MF (76.80 ± 8.52 mg/kg) > EF (64.05 ± 8.97 mg/kg). The AP content of the surface soil (0–25 cm) in EF (1.20 ± 0.11 mg/kg) was significantly higher than that in other forests (0.58 ± 0.08 mg/kg in MF and 0.54 ± 0.10 mg/kg in LF, respectively; Figure 3B). The AP content for the 0–100 cm depth decreased from 0.64 ± 0.08 mg/kg (EF) to 0.39 ± 0.04 mg/kg (LF). As shown in Figure 3C, the AK content showed a decreasing trend with increasing soil depth in each forest, the average value of which was significantly decreased in the order EF (75.53 ± 6.81 mg/kg) > MF (49.13 ± 6.39 mg/kg) > LF (26.45 ± 3.73 mg/kg) for the 0–100 cm depth.

### Differences in the Soil Stoichiometric Characteristics of the Three Forests

**Figure 4:**
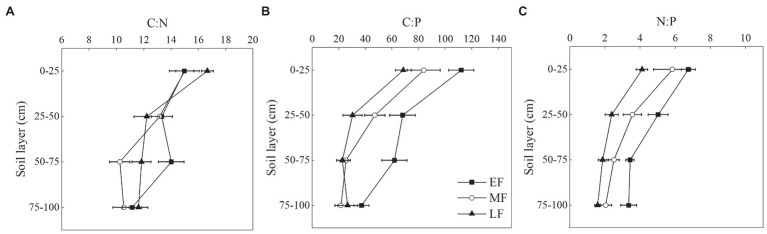
Vertical changes of C:N **(A)**, C:P **(B)**, and N:P **(C)** in the 0–100 cm soil layer of different forests. Values are the means ± SE (*n* = 6). C:N, SOC:TN; C:P, SOC:TP; N:P, TN:TP; EF, broadleaf–conifer mixed forest; MF, broad-leaved forest; LF, old-growth forest.

As shown in Figures 4A,B, the C:N and C:P showed an overall decreasing trend in the vertical soil profile in each forest. There was no significant difference in C:N at the depth of 0-100 cm between the three forests, which were 13.36 ± 0.88(EF), 12.23 ± 0.79(MF), 13.08 ± 0.69(LF). The C:P in the 0–25 cm soil layer was significantly higher than that in other soil layers (Figure 4B). When averaged across the 0–100 cm depth, the C:P decreased from 72.33 ± 6.90 (EF) to 37.68 ± 4.76 (LF). As shown in Figure 4C, the average N:P differed significantly between forests, that is, EF (4.64 ± 0.35) > MF (3.49 ± 0.42) > LF (2.49 ± 0.24).

**Figure 5:**
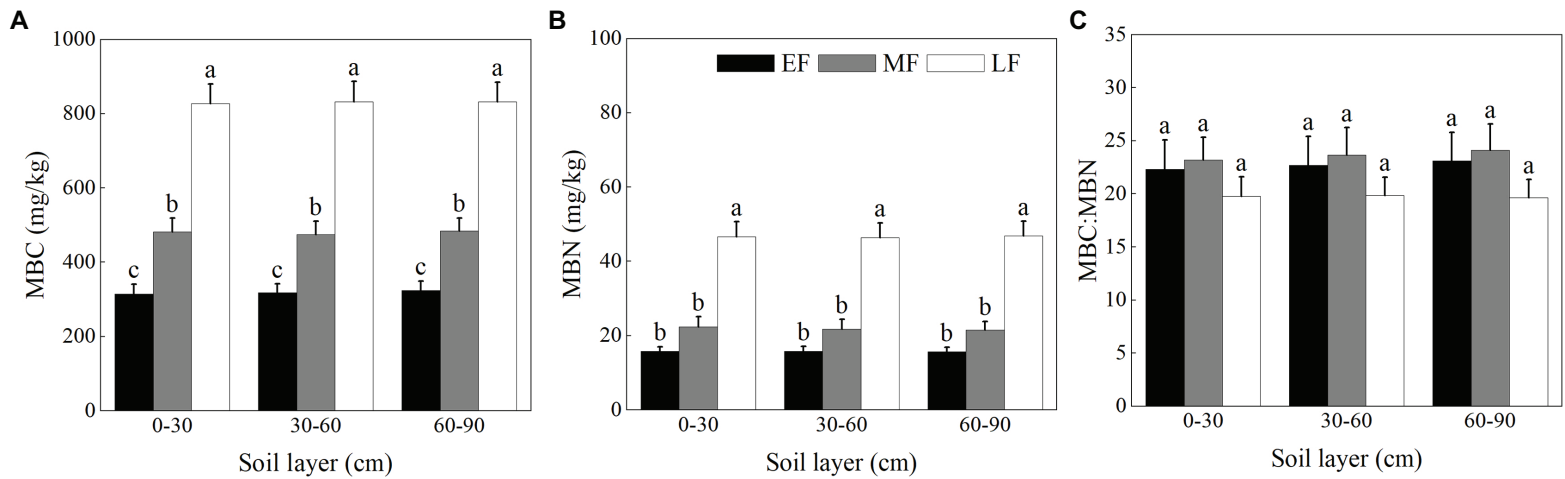
The contents of MBC **(A)**, MBN **(B)**, and the ratio of MBC:MBN **(C)** in the 0–100 cm soil layer in different forests. Values are the means ± SE (*n* = 6). Lowercase letters above the columns represent statistically significant differences among stand types for the same soil layer (Duncan’s tests, *p* < 0.05). MBC, soil microbial biomass C; MBN, soil microbial biomass N; EF, broadleaf–conifer mixed forest; MF, broad-leaved forest; LF, old-growth forest.

As shown in Figure 5A, the MBC did not differ significantly among soil layers in each forest. Within the 0–90 cm soil layer, there was a significant difference in the MBC between forests, that is, LF (830.34 ± 30.34 mg/kg) > MF (480.36 ± 19.77 mg/kg) > EF (319.03 ± 14.24 mg/kg). As shown in Figure 5B, the MBN for the 0–90 cm depth was significantly higher in LF (46.60 ± 2.27 mg/kg) than in other forests (15.78 ± 0.68 mg/kg in EF and 21.90 ± 1.41 mg/kg in MF, respectively). In each forest, the MBC:MBN was not significantly different between layers. When averaged across the 0–90 cm depth, there was no significant difference in the MBC:MBN between forests, that is, LF (19.75 ± 1.00) < EF (22.71 ± 1.55) < MF (23.66 ± 1.33) (Figure 5C).

### The Relationship Between Soil Properties and Comprehensive Evaluation

**Figure 6:**
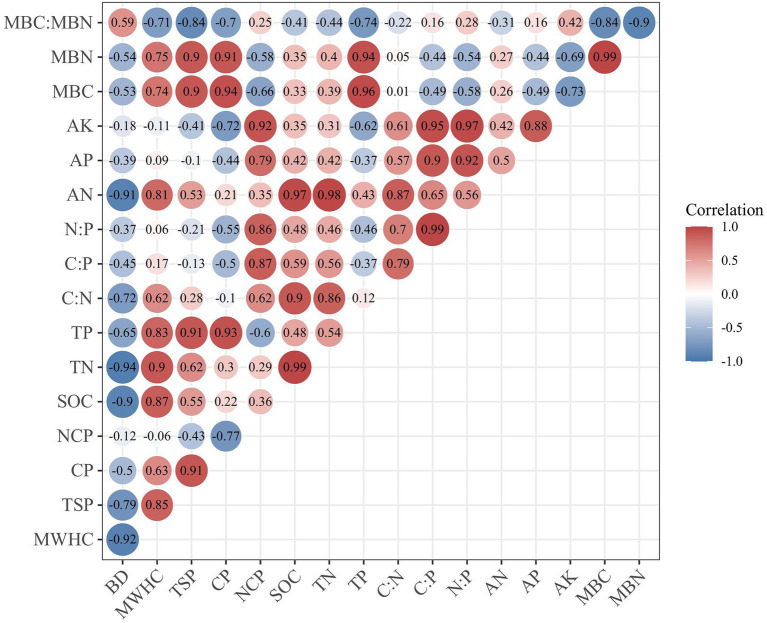
Pearson’s correlation coefficients among soil properties. BD, bulk density; MWHC, maximum soil water holding capacity; TSP, total soil porosity; CP, capillary porosity; NCP, non-capillary porosity; SOC, soil organic carbon; TN, soil total nitrogen; TP, soil total phosphorus; AN, alkali-hydrolyzable nitrogen; AP, available phosphorous; AK, available potassium; MBC, soil microbial biomass C; MBN, soil microbial biomass N.

Figure 6 shows the relationship between different soil properties. The BD was negatively correlated with TSP (−0.79, *p* < 0.05) and SOC (−0.90, *p* < 0.01). MWHC was mainly affected by TSP (0.85, *p* < 0.01) and SOC (0.87, *p* < 0.01). The SOC was closely related to TN (0.99, *p* < 0.01) and AN (0.97, *p* < 0.01). The physical properties of soil also have a certain impact on the stoichiometric characteristics. The NCP was positively correlated with C:P (0.87, *p* < 0.01) and N:P (0.86, *p* < 0.01). As for soil microbial biomass C and N, the TP was positively correlated with MBC and MBN, and the correlation coefficients were 0.96 and 0.94, respectively (*p* < 0.01). While the content of AK was negatively correlated with MBC and MBN, and the correlation coefficients were −0.73 and −0.69, respectively (*p* < 0.05).

A comprehensive index, that is, SQI was established to evaluate the change in soil properties. Based on the principal component analysis and the correlation between various properties, a soil quality index equation was constructed. The principal component analysis showed that values of the first two PCs were all ≥1 and explained 94.22% of the total variance. TSP, TP, and MWHC in the first principal component (PC1) are high-weight indicators (Appendix 1). MWHC had a high correlation with TSP and TP (Figure 6), and the load coefficients of TSP and TP were the same (Appendix 1), so TSP and TP were retained in PC1. In the second principal component (PC2), AP and AK were highly weighted factors and are significantly correlated with each other. AK was selected in PC2 because AK had a higher load factor (Appendix 1). Finally, we selected TSP, TP, and AK in MDS to calculate SQI. With the weighting values based on PCA (Appendix 2), the calculation of SQI was given by Eq. (5):


(5) SQI=0.61TSP+0.61TP+0.39AK

**Figure 7:**
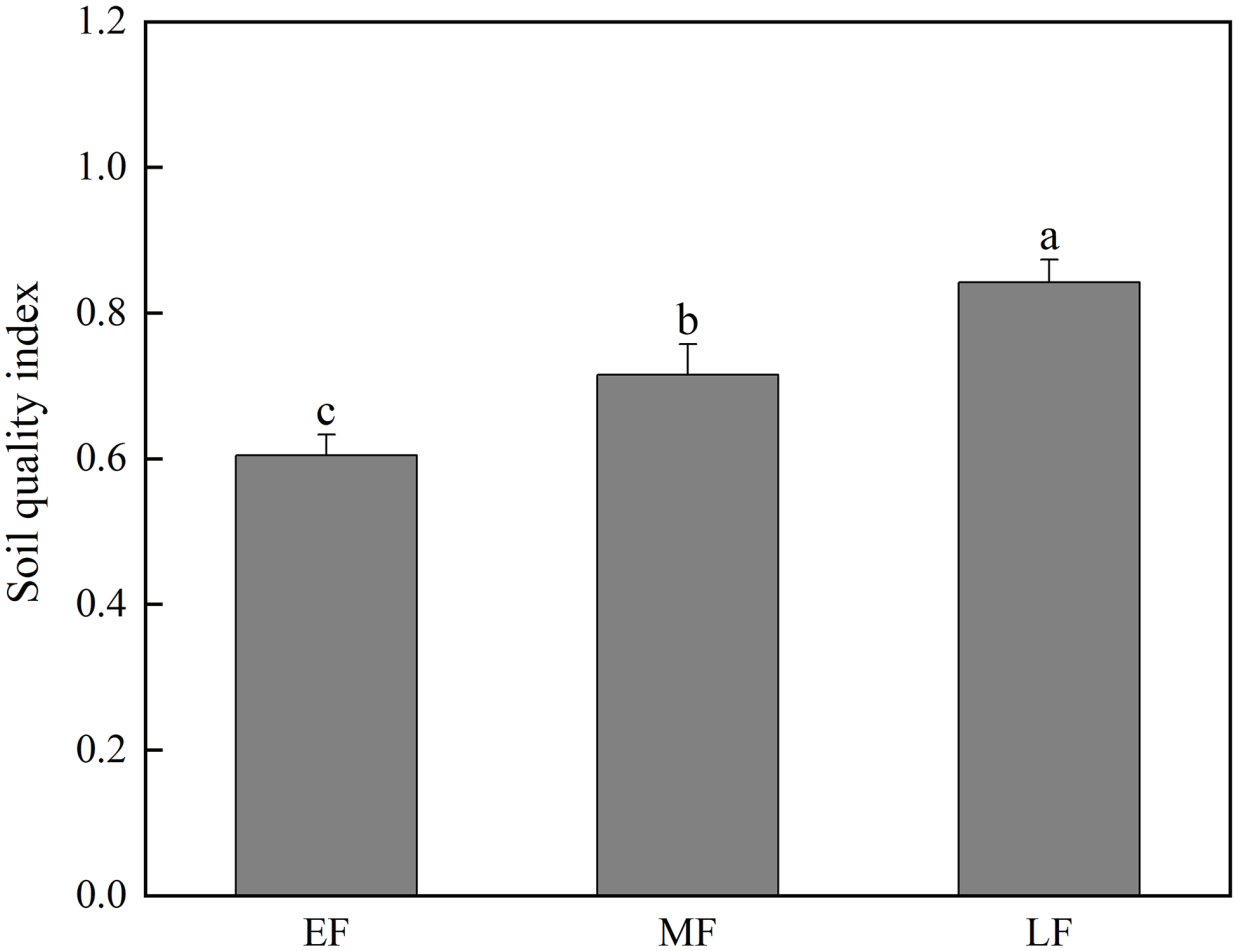
The 0–100 cm soil quality index under different forests types. Values are the means ± SE (*n* = 6). The lowercase letters above the column represent the statistically significant differences between different stand types (Duncan’s tests, *p* < 0.05). EF, broadleaf–conifer mixed forest; MF, broad-leaved forest; LF, old-growth forest.

According to the calculation results of Eq. (5), the soil quality had been improved, respectively, during the forest restoration [(*p* < 0.05), that is, LF (0.84) > MF (0.72) > EF (0.60; Figure 7)].

## Discussion

### Changes in Soil Physico-Chemical Properties During Forest Restoration

In this study, the SOM increased along forest succession, consistent with the results of Huang et al. (2018) and Bu et al. (2018). The increase of SOM promotes the growth of roots and the activity of soil microorganisms, which is conducive to the development of soil pores and the formation of soil aggregates. In addition, the chemical conditions created by the secretion of organic acids by roots, microorganisms, and organic matter decomposition also promote the fracturing of soil particles, which eventually leads to the decrease of BD and the improvement of MWHC (Marschner et al., 2011; Liu et al., 2019; Pastore et al., 2020). In the vertical soil profile of different forests, the BD in the surface layer was significantly lower than that in other soil layers. This is because litter decomposition inputs a large amount of soil organic matter into the surface layer, which improves the surface soil structure (Porter et al., 2010; Freschet et al., 2013). Secondly, the increased density of deep soil was caused by the compaction of the soil by root growth (Shah et al., 2017). The combined action of these two processes led to a state of loose soil in the upper layer and compaction in the lower layer.

Vegetation types can significantly affect the soil’s physical and chemical properties (Srivastava et al., 2020; Wang et al., 2020). The research showed that there were significant differences in soil nutrient characteristics among the three forests. The SOC, TN, and TP were significantly higher in the late stage of forest restoration than that in other stages, indicating that vegetation restoration was conducive to the improvement of the soil carbon sink effect in south China (Hu et al., 2021). This phenomenon has been confirmed by a large number of studies (Jiang et al., 2017; Zhang et al., 2018). The vegetation provides the carbon and energy sources for the soil through root exudates and plant residues (Zhu et al., 2010) which may be related to the plant diversity and species turnover (Smith et al., 2002). As positive forest succession progresses, the increasing amounts of litter return result in higher content of SOM (Bu et al., 2018). Thereafter, root growth is promoted and fine root biomass increases (Feng et al., 2019), thus the SOC and TN increase significantly. It has been reported that the SOC played a key role in the availability of soil N and P, which could be explained in two aspects (Liu et al., 2021). On the one hand, the SOC could reduce the adsorption of N and P by competing with soil exchangeable base cations for adsorption sites (Schreeg et al., 2013; Liu et al., 2021). On the other hand, the SOC enhances the activity of soil microorganisms by providing energy, potentially promoting soil N and P mineralization (Lange et al., 2015; Chen et al., 2019b). The vertical distribution pattern of forest soil nutrients is related to its source (Bai et al., 2019). Our results showed that the SOC and TN were concentrated on the soil surface with dense roots, which were conducive to the absorption of nutrients by plants. This was consistent with the study by Liu et al. (2020). The content of the TP in each soil layer does not change by much in our study. A possible explanation for this was that the P was extracted from the soil parent material as a consequence of mainly weathering and biology. In addition, the growing forests satisfied the increasing nutrient demand by extending their roots deeper into the soil, which also reduced the spatial heterogeneity of P (Guo et al., 2021; Pastore et al., 2022).

The total amount of soil nutrients reflects the capacity of nutrient storage, while the available nutrient content mirrors the validity of nutrients. Analysis data indicated that the AN showed an increasing trend with the chronosequence, while the AP and AK showed an opposite trend. Researchers proposed that the concentrations of soil available nutrients (i.e., AN, AP, and AK) were species-dependent, which could be explained by the “complementary” or “mass” effects related to plant diversity in forest ecosystems (Zheng et al., 2017; Liu et al., 2021). Han et al. (2021) demonstrated that the content of AK showed a positive correlation with BD. In LF, BD decreased while nutrient leaching increased, which may also be the reason for the decline in soil AK content with forest succession. In this study, different kinds of soil available nutrients in each forest showed a downward trend in the profile. According to the results of correlation analysis, the AN showed a significantly positive relationship with MWHC (0.81) and SOC (0.97), consistent with the results of Gairola et al. (2012), which could be attributed to the patterns of litter return and water distribution. The AP (0.79) and the AK (0.92) showed a significantly positive relationship with NCP. The leaching properties of the soils might dominate the distribution of AP and AK among layers.

### Changes in Soil Stoichiometric Ratio During Forest Restoration

Soil stoichiometry is the core of C, N, and P biogeochemistry (Sardans and Peñuelas, 2015), which regulates the aboveground and underground nutrient cycle of forest ecosystems (Güsewell and Gessner, 2009). In this study, soil C:N had no significant difference among forests, but the C:P and N:P significantly decreased alongside forest restoration. This situation might be due to the high level of atmospheric acid deposition in south China. In the early stage of forest restoration in a broadleaf–conifer mixed forest, the precipitation interception effect of the forest canopy was lower than that of the broad-leaved forest therefore, more acid rain fell on the soil surface (Li et al., 2015). Nitrogen (N) is one of the main elements that get deposited in the process of atmospheric acid deposition. The combination of exogenous N and acid deposition exacerbates the limitation of soil P (Tie et al., 2020). In the case of P limitation, microorganisms and plants usually secrete more phosphatase, which catalyzes the mineralization and decomposition of organic phosphorus to produce AP (Liu et al., 2014). Therefore, the content of AP in the early stage of forest restoration was higher than that in the late stage of forest restoration.

Along the soil vertical profile in this study, the stoichiometric ratio in the surface layer of different forests was higher than that in other soil layers. Subtropical China is one of the regions with the highest level of nitrogen deposition in the world (Liu et al., 2011). The increase of soil acidity under N deposition inhibits soil respiration by lowering bacterial and fungal biomass and proteolytic enzyme activity, which weakens the rates of litter decomposition and the ability of the microbial community to utilize a suite of C substrates, then the accumulation of soil carbon promotes (Mo et al., 2008). This phenomenon may further increase the C:N of surface soil and reduce the C:N of deep soil (Yu et al., 2017). Nitrogen deposition also promotes P absorption and plant growth (Deng et al., 2016; Li et al., 2016). In the soil surface layer with a dense root distribution, the decline of soil P concentration is more serious than that in the lower layer.

### Changes in Soil Microbial Biomass C and N During Forest Restoration

Soil microbial biomass carbon (MBC) and microbial biomass nitrogen (MBN) are the main components of soil microbial biomass, and their subtle changes affect the turnover of the soil organic matter and nutrients (Yang et al., 2010). In this study, the contents of the MBC and the MBN increased significantly alongside forest restoration, and the MBC:MBN between forests was not significant. Vivanco and Austin (2008) reported that litter quality can affect microbial growth and reproduction by affecting microbial substrate decomposition and altering decomposition difficulty. Compared to the broad-leaved litter in the later stage of forest restoration, the contents of lignin, terpenoids, and phenols in the litter of the broadleaf–conifer mixed forest were more difficult to decompose, and the effectiveness of microbial substrate was lower. What’s more, the microbial groups and quantities that could be supported were relatively small in the broadleaf–conifer mixed forest (Chai et al., 2019; Yang et al., 2020). Therefore, the contents of the MBC and the MBN were relatively low in the early stage of forest restoration. In the vertical profile of soil, there was no significant difference in the MBC and the MBN contents among layers in each forest. Atmospheric acid deposition and litter in the subtropical forests of southern China provided a large amount of N and P elements to the soil. According to the results of the correlation analysis, the effect of N increment on the MBC of subtropical soils was not significant, and the soil microbial biomass was significantly affected by TP, which was consistent with Chu et al. (2007) P comes from the differentiation of the soil parent rock. In our study, the differences in P between different soil layers in each forest were not significant, which might be the reason for the insignificant difference in the contents of the MBC and the MBN between layers.

Soil microbial biomass is very sensitive to changes in soil environmental factors (Zhao et al., 2009). Hu et al. (2016) proposed that the improvement of soil porosity and moisture was the main reason for the increase in microbial biomass and activity. Larger soil porosity would make soil aeration better, which could provide optimal environmental conditions for soil microorganisms (Marinari et al., 2000). In this study, it was also reported that the contents of MBC (0.94, 0.90) and MBN (0.91, 0.90) were significantly positively correlated with soil capillary porosity and total porosity. Researchers proposed that larger soil porosity always indicated a stronger capacity of soil potential water conservation (Marinari et al., 2000). A synergistic relationship between soil microorganisms and soil moisture has been confirmed by a large number of studies (Kaneda and Kaneko, 2011; Borowik and Wyszkowska, 2016; Hawkes et al., 2017). In our study, the contents of the MBC (0.74) and the MBN (0.75) were positively correlated with the MWHC. As a critical carrier of material cycle and energy exchange, soil moisture plays an important role in the hydrological processes and nutrient circulation of forest ecosystems (Laclau et al., 2013). Large amounts of soil water would transport or diffuse soil nutrients to places where microorganisms need it (Illeris et al., 2003), thus microbial biomass became more abundant in pace with the soil water content improvement (Zhang et al., 2005; Baldrian et al., 2010). On the contrary, low soil water content would limit the mobility of nutrients and microbial activity.

### Changes in Soil Quality Index During Forest Restoration

Based on the basic properties of soil, a comprehensive index (SQI) was constructed to reflect the changes in soil under forest restoration, which is of great significance to evaluate sustainable land use and effective management (Zhang et al., 2019a). In this study, 13 indexes based on soil microbial biomass and soil physico-chemical properties were selected, and MDS was selected by principal component analysis to evaluate soil quality. From the evaluation results, the soil quality in this area is relatively good, and with the forest restoration, the soil quality has improved to a certain extent, which is consistent with the previous research results (An et al., 2009; Yu and Jia, 2014; Guo et al., 2018). In our study, TSP and TP contributed the most to the comprehensive SQI, indicating that TSP and TP played an important role in determining soil quality. As an important index of soil nutrient content, TP can directly reflect soil fertility. P availability also further determines plant nutrient constraints and can affect processes important to ecosystem function (Guo et al., 2018). As an important physical index of soil, TSP can directly reflect the aeration, water storage, and hydraulic conductivity of soil (de Moraes et al., 2019). Therefore, it is very reasonable to select these two indicators in our MDS to reflect soil quality. In our study, due to the increase of litterfall production and turnover rate in the later stage of forest restoration, TSP and TP were improved, which further led to the increase in soil quality.

## Conclusion

Our findings show that secondary forest restoration in southern China significantly affected soil physico-chemical and microbiological properties. As forest restoration proceeded, soil properties, that is, BD, porosity, and water holding capacity were significantly improved. The soil structure especially the topsoil was also optimized to a great extent. Total soil nutrients and soil microbial biomass gradually increased, while the amounts of the available nutrients, that is, AP and AK showed a downward trend. In addition to TP, there was a significant surface enrichment of forest soil nutrients. The SQI, which integrated physico-chemical and microbiological properties, demonstrated that higher soil quality was mainly affected by soil porosity and total phosphorus in this area. The SQI as a forest restoration evaluation method proved to be effective in revealing the local factors that require more attention for sustainable and scientific forestry management. The present study provides new evidence that soil properties improved during the restoration of a secondary subtropical forest, and is of great significance for evaluating the function and improving the management scheme of subtropical forests in South China.

## Data Availability Statement

The datasets generated and analyzed during the current study are available from the corresponding authors on reasonable request.

## Author Contributions

XZ, PL, and YF: data processing and writing. WZ: conceptualization and data collection. BN: language correction. FL, QZ, CQ, and XG: site maintenance and data collection. XL: experimental design and review. All authors contributed to the article and approved the submitted version.

## Funding

This work was supported by the Forestry Science and Technology Innovation Project of Guangdong Province, China (2021KJCX003 and 2022KJCX015) and Operation Subsidy Program of Technology Innovation Platform of National Forestry and Grassland Administration (2021132084 and 2021132085).

## Conflict of Interest

The authors declare that the research was conducted in the absence of any commercial or financial relationships that could be construed as a potential conflict of interest.

## Publisher’s Note

All claims expressed in this article are solely those of the authors and do not necessarily represent those of their affiliated organizations, or those of the publisher, the editors and the reviewers. Any product that may be evaluated in this article, or claim that may be made by its manufacturer, is not guaranteed or endorsed by the publisher.

## Supplementary Material

The Supplementary Material for this article can be found online at:

https://www.frontiersin.org/articles/10.3389/fpls.2022.878908/full#supplementary-material
