# Supplementary material for: Changes in Soil Physico-Chemical and Microbiological Properties During Natural Succession: A Case Study in Lower Subtropical China
Source: Front Plant Sci. 2022 Jun 3;13:878908. doi: 10.3389/fpls.2022.878908 (PMC9204105; doi:10.3389/fpls.2022.878908)
Supplement: Supplementary file 1 [file Data_Sheet_1.docx]

**Appendix 1.** Principal component analysis of soil quality indicators.

| Indicator | Principal component | |
| --- | --- | --- |
|  | **PC1** | **PC2** |
| Eigenvalues | 7.485 | 4.763 |
| Variance (%) | 57.580 | 57.580 |
| Cumulative (%) | 36.640 | 94.220 |
| BD | -0.818 | -0.546 |
| MWHC | **0.931** | 0.321 |
| TSP | **0.962** | -0.050 |
| CP | 0.865 | -0.436 |
| NCP | -0.400 | 0.858 |
| SOC | 0.662 | 0.712 |
| TN | 0.718 | 0.680 |
| TP | **0.962** | -0.239 |
| AN | 0.625 | 0.749 |
| AP | -0.162 | **0.876** |
| AK | -0.410 | **0.901** |
| MBC | 0.912 | -0.386 |
| MBN | 0.906 | -0.342 |

**Abbreviations**: Bold factors are considered highly weighted; underlined and bold factors are retained in the minimum data set (MDS). PC1, PC2 indicate the first principal component, second principal component, third principal component, and fourth principal component, respectively. BD, *bulk density*; MWHC, *maximum soil water holding capacity*; TSP, *total soil porosity*; CP, *capillary porosity*; NCP, *noncapillary porosity*; SOC, *soil organic carbon*; TN, *soil total nitrogen*; TP, *soil total phosphorus*; AN, *alkali-hydrolyzable nitrogen*; AP, *available phosphorous*; AK, *available potassium*; MBC, *soil microbial biomass C*; MBN, *soil microbial biomass N.*

**Appendix 2:** Normalization equation of scoring curves.

| Parameter | TSP | TP | AK |
| --- | --- | --- | --- |
| Average(x_0_) | 0.46 | 0.18 | 55.08 |
| Curve type | More is better | More is better | More is better |
| Slope(b) | -2.5 | -2.5 | -2.5 |
| Normalization equation | S=1/(1+(x/0.46) ^(-2.5)^) | S=1/(1+(x/0.18)^(-2.5)^) | S=1/(1+(x/55.08)^(-2.5)^) |
| Weighting value | 0.61 | 0.61 | 0.39 |

**Abbreviations**: TSP, total soil porosity; TP, soil total phosphorus; AK, available potassium.
